# Wearable systems for shoulder kinematics assessment: a systematic review

**DOI:** 10.1186/s12891-019-2930-4

**Published:** 2019-11-15

**Authors:** Arianna Carnevale, Umile Giuseppe Longo, Emiliano Schena, Carlo Massaroni, Daniela Lo Presti, Alessandra Berton, Vincenzo Candela, Vincenzo Denaro

**Affiliations:** 1 Department of Orthopaedic and Trauma Surgery, Campus Bio-Medico University, Via Álvaro del Portillo, 200, 00128 Rome, Italy; 2 Unit of Measurements and Biomedical Instrumentation, Campus Bio-Medico University, Via Álvaro del Portillo, 21, 00128 Rome, Italy

**Keywords:** Shoulder kinematics, Upper limb, Wearable system, Inertial sensors, Smart textile

## Abstract

**Background:**

Wearable sensors are acquiring more and more influence in diagnostic and rehabilitation field to assess motor abilities of people with neurological or musculoskeletal impairments. The aim of this systematic literature review is to analyze the wearable systems for monitoring shoulder kinematics and their applicability in clinical settings and rehabilitation.

**Methods:**

A comprehensive search of PubMed, Medline, Google Scholar and IEEE Xplore was performed and results were included up to July 2019. All studies concerning wearable sensors to assess shoulder kinematics were retrieved.

**Results:**

Seventy-three studies were included because they have fulfilled the inclusion criteria. The results showed that magneto and/or inertial sensors are the most used. Wearable sensors measuring upper limb and/or shoulder kinematics have been proposed to be applied in patients with different pathological conditions such as stroke, multiple sclerosis, osteoarthritis, rotator cuff tear. Sensors placement and method of attachment were broadly heterogeneous among the examined studies.

**Conclusions:**

Wearable systems are a promising solution to provide quantitative and meaningful clinical information about progress in a rehabilitation pathway and to extrapolate meaningful parameters in the diagnosis of shoulder pathologies. There is a strong need for development of this novel technologies which undeniably serves in shoulder evaluation and therapy.

## Background

Shoulder kinematics analysis is a booming research field due to the emergent need to improve diagnosis and rehabilitation procedures [1]. The shoulder complex is the human joint characterized by the greatest range of motion (ROM) in the different planes of space.

Commonly, several scales and tests are used to evaluate shoulder function, e.g., the Constant-Murley score (CMS), the Simple Shoulder test (SST), the Visual Analogue Scale (VAS) and the Disability of the Arm, Shoulder, and Hand (DASH) score [2–4]. However, despite their easy-to-use and wide application in clinical settings, these scores conceal an intrinsic subjectivity [2–4], inaccuracy in approaching diagnosis, follow-up and treatment of the pathologies. Quantitative and objective analyses are rapidly developing as a valid alternative to evaluate shoulder activity level, to gauge its functioning and to provide information about movement quality, e.g., velocity, amplitude and frequency [5, 6]. This interest in the use of measuring systems is growing in many medical fields to record information of clinical relevance. For example, electromyography (EMG), force sensors, inertial measurement units (IMU), accelerometers, fiber optic sensors and strain sensors are employed for human motion analysis, posture and physiological parameters monitoring [7–10]. From a technological viewpoint, the monitoring of shoulder motion is challenging due to the complexity of joint kinematic which require the development of protocols exploiting sensing technology as much as possible reliable and unobtrusive. In the last years, a great number of human motion analysis systems have been largely employed for objective monitoring. These systems can be classified into two main categories: wearable and non-wearable [11]. The last one includes electromagnetic tracking systems (e.g., Fastrak) [12], ultrasound-based motion analysis systems (e.g., Zebris) [13], stereo-photogrammetric and optoelectronic systems (e.g., VICON, Optotrak, BTS SMART-D) often used as gold standard [14–17]. These systems based on magnetic field, ultrasound and cameras are effectively suitable for 3D motion tracking and analysis due to their accuracy, precision and reliability [18]. On the other hand, such systems require expensive equipment, frequent calibration and, overall, they restrict measurements in structured environment [19]. Wearable systems overcome these shortcomings and they are a promising solution for continuous and long-term monitoring of human motion in daily living activities. Gathering data in unstructured environment continuously (e.g., home environment) provide additional information compared to those obtainable inside a laboratory [20].

Wearable sensor-based systems, intended for kinematics data extraction and analyses, are acquiring more and more influence in diagnostic applications, rehabilitation follow-up, and treatments of neurological and musculoskeletal disorders [21, 22]. Such systems comprise accelerometers, gyroscopes, IMU, among others [23]. Patients’ acceptance of monitoring systems that should be worn for long-time relies on sensors’ features whose must be lightweight, unobtrusive and user-friendly [24]. The increasing trend to adopt such wearable systems has been promoted by the innovative technology of micro-electro-mechanical systems (MEMS). MEMS technology has fostered sensors’ miniaturization, paving the way for a revolutionary technology suited to a wide range of applications, including extraction of clinical-relevant kinematics parameters. In recent years, there has been growth in the use of smart textile-based systems which integrate sensing units directly into garments [11, 25, 26]. Moreover, in the era of big data, machine learning technical analysis can improve home rehabilitation thanks to the recognition of the quality level of performed physical exercises and the possibility to prevent disorders in patients’ movement [27].

The aim of this systematic literature review is to describe the wearable systems for monitoring shoulder kinematics. The authors want to summarize the main features of the current wearable systems and their applicability in clinical settings and rehabilitation for shoulder kinematics assessment.

## Methods

### Literature search strategy and study selection process

A systematic review was executed applying the PRISMA (Preferred Reporting Items for Systematic Reviews and Meta-Analyses) guidelines [28]. Full-text articles and conference proceedings were selected from a comprehensive search of PubMed, Medline, Google Scholar and IEEE Xplore databases. The search strategy included free text terms and Mesh (Medical Subject Headings) terms, where suited. These terms were combined using logical Boolean operators. Keywords and their synonyms were combined in each database as follows: (“shoulder biomechanics” OR “upper extremit*” OR “shoulder joint” OR “scapular-humeral” OR “shoulder kinematics” OR “upper limb”) AND (“wearable system*” OR “wearable device*” OR “wearable technolog*” OR “wearable electronic device*” OR “wireless sensor*” OR “sensor system” OR “textile” OR “electronic skin” OR “inertial sensor”). No filter was applied on the publication date of the articles, and all results of each database were included up to July 2019. After removal of duplicates, all articles were evaluated through a screening of title and abstract by three independent reviewers. The same three reviewers performed an accurate reading of all full-text articles assessed for eligibility to this study and they performed a collection of data to minimize the risk of bias. In case of disagreement among investigators regarding the inclusion and exclusion criteria, the senior investigator made the final decision.

Inclusion criteria were:

i) The studies concern wearable systems as a tool to assess upper limb kinematics;
ii) The studies used sensors directly stuck on the human skin by means of adhesive, embedded within pockets, straps or integrated into fabrics;
iii) Systems intended for motion recognition and rehabilitation;
iv) Articles are written in English language;
v) Papers are published in a peer-reviewed journal or presented in a conference;

Exclusion criteria were:

i) Use of prosthetics, exoskeleton or robotic systems;
ii) Wearable system not directly worn or tested on human;
iii) The study concerns wearable systems for full-body motion tracking;
iv) Shoulder joint is not included;
v) Reviews, books.

### Data extraction process

Data extraction was executed on 73 articles. Data was extracted on the base of the following checklist: authors, year and type of publication (i.e., conference or full-text); typology, number, brand and placement of the sensors used to measure or track the kinematic of the interested joint, wearability of the system, target parameters with regard to the shoulder; system used as gold standard to assess the wearable systems’ performance; tasks executed in the assessment protocol; characteristics of the participants involved in the study and aim of the study.

## Results

**Fig. 1:**
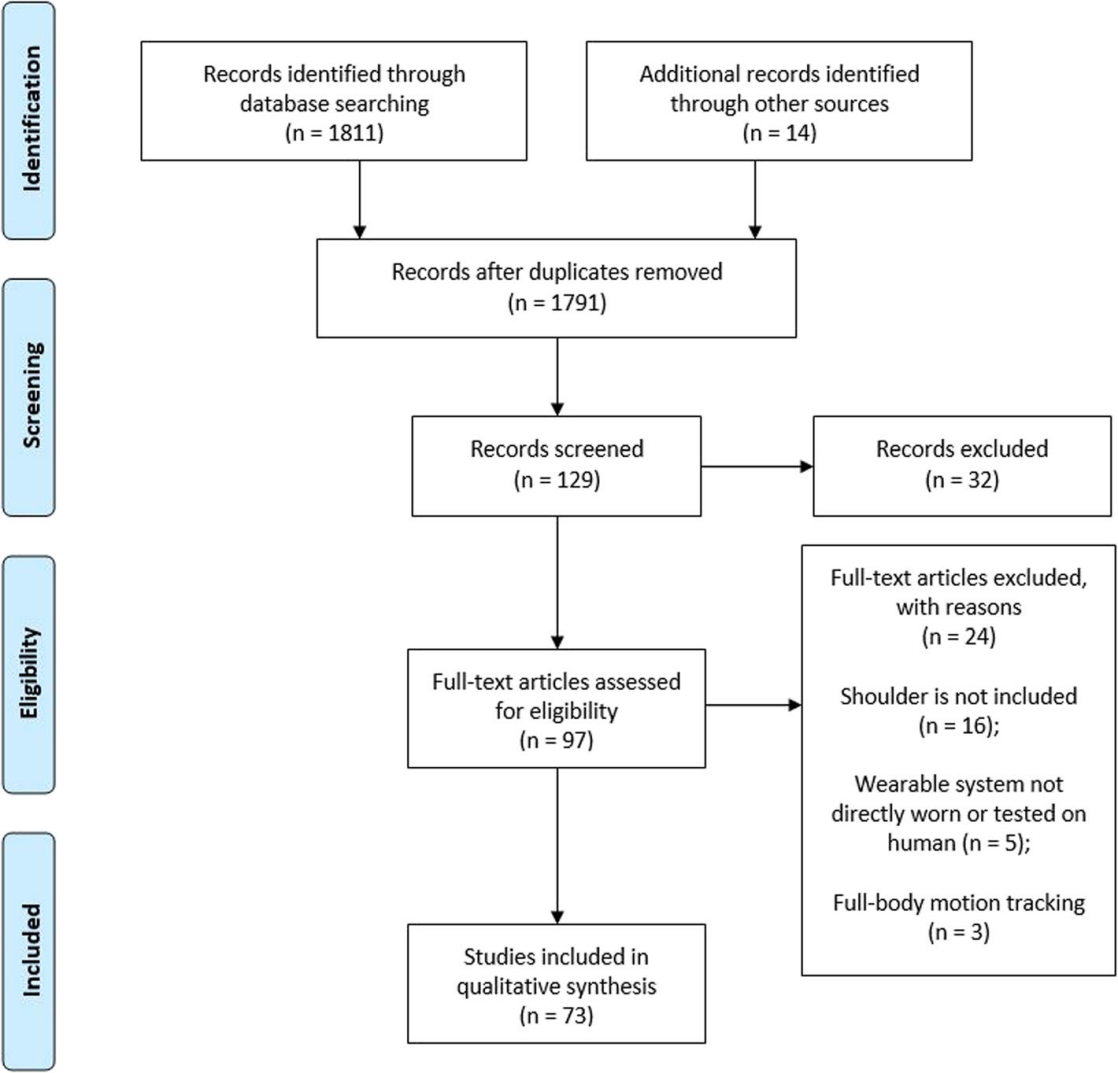
PRISMA 2009 flow diagram

The literature search returned 1811 results and additional 14 studies were identified through other sources. A total of 73 studies fulfilled the inclusion criteria (Fig. 1), of which 27% were published on conference proceedings and the remaining 73% on peer-reviewed journal.

Three levels of analysis have been emphasized in this survey: A. application field and main aspects covered, B. the typology of sensors exploited to measure kinematic parameters, C. the placement of the single measurement units on the body segment of interest and how sensing modules are integrated into the wearable system from a wearability viewpoint.

### Application field

Fifteen out of the 73 studies focused on evaluating upper limbs motion in case of musculoskeletal diseases (e.g., osteoarthritis, rotator cuff tear, frozen shoulder), 26 on neurological diseases and ap-plication in neurorehabilitation (e.g., stroke, multiple sclerosis), 15 on general rehabilitation aspects (e.g., home rehabilitation, physiotherapy monitoring) and 17 focusing on validation and development of systems and algorithm for monitoring shoulder kinematics. Tables 1, 2, 3 and 4 include, for each of the identified application fields, data listed in the previous data extraction process section.

**Table 1:** Shoulder motion monitoring for application in patients with musculoskeletal disorders

Reference, Year, Type of publication	Sensors, Brand	Placement and wearability	Target shoulder parameters, Performance	Gold standard	Task executed	Participants	Aim
Coley 2007, [29] Full-Text	IMU (*n* = 2), Analog Device (gyr: ADXRS 250, acc: ADXL 21 0)	Bilateral: humeri (posteriorly, distally) Adhesive patch	Sh ROM (HT joint angles), RMSE =5.81° Mean = 1.80°	Zebris CMS-HS	Sh FLX-EXT Sh AB-AD Sh IER	HS (*n* = 10), 25.1 ± 4.1 Y P (*n* = 10), 7 RC, 3 OA, 4 F, 6 M, 62.4 ± 10.4 Y	Find objective scores to assess shoulder function; to validate such scores comparing healthy and affected shoulders
Coley 2008, [30] Full-Text	IMU (*n* = 4), Analog Device (gyr: ADXRS 250, acc: ADXL 21 0)	Bilateral: humeri (posteriorly, distally), thorax Adhesive patch	Sh ROM (HT joint angles)	–	Daily activities (8 h)	HS (*n* = 35) 32 ± 8 Y	Quantify usage of shoulder and the contribution of each shoulder in daily life activities
Jolles 2011, [31] Full.Text	IMU (*n* = 4), Analog Device (gyr: ADXRS 250, acc: ADXL 21 0)	Bilateral: Humeri (distally, posteriorly), thorax (× 2) Adhesive patch	Sh ROM (HT joint angles), r = 0.61–0.80	–	7 activities of SST	P (*n* = 34) 27 RC, 7 OA 25 M, 9 F 57.5 ± 9.9 CG (*n* = 31) 17 M, 14 F 33.3 ± 8.0	Validate clinically shoulder parameters in patients after surgery for GH OA and RC disease
Duc 2013, [5] Full-Text	IMU (*n* = 3), Analog Device (gyr: ADXRS 250, acc: ADXL 210)	Bilateral: Humeri (distal, posterior), sternum Adhesive patch	Sh ROM (HT joint angles), *r*^2^ = 0.13-0.52 (CMS) *r*^2^ = 0.06-0.30 (DASH) *r*^2^ = 0.03-0.31 (SST)	–	Free movements, daily activities	HS (*n* = 41) 34 ± 9 Y P (*n* = 21) 53 ± 9 Y RC (unilateral)	Validate a method to detect movement of the humerus relative to the trunk and to provide outcomes parameters after shoulder surgery (frequency of arm movement and velocity)
Körver 2014, [32] Full-Text	IMU (*n* = 2), Inertia-Link-2400-SKI, MicroStrain	Bilateral: Humerus (distal, posterior) Adhesive	Sh ROM (HT joint angles), r = 0.39 (DASH) r = 0.32 (SST)	–	Hand to the back, Hand behind the head	HS (*n* = 100) 37 M, 63 F 40.6 ± 15.7 Y P (*n* = 15) 5 M, 10 F 57.7 ± 10.4 Y, Subacromial impingement	Investigate about the correlation between subjective (clinical scale) and objective (IMU) assessment of shoulder ROM during a long-term period of follow-up
Van Den Noort 2014, [33] Full-Text	M-IMU (*n* = 4), Xsens MTw	Unilateral: Thorax, scapula, UA, FA Straps, skin tape	Sh ROM (HT and ST joint angles)	–	FLX (sagittal plane) and AB (frontal plane) with elb extended and thumb pointing up	HS (*n* = 20) 3 M, 17 F 36 ± 11 Y	Evaluate the intra- and inter-observer reliability and precision of 3D scapula kinematics
Pichonnaz 2015, [34] Full-Text	IMU (*n* = 3), Analog Device (acc: ADXL 210, gyr: ADXRS 250)	Bilateral: Sternum, Humeri (posterior, distal) Skin tape	Sh ROM (HT joint angles)	–	Free movements (7 h)	HS (*n* = 41) 23 M, 18 F 34.1 ± 8.8 Y P (*n* = 21) 14 M, 7 F 53.3 ± 9 Y RC	Explore dominant and non-dominant arm usage as an indicator of UL function after rotator cuff repair during the first year after surgery
Roldán-Jiménez e Cuesta-Vargas 2015, [35] Full-Text	M-IMU (*n* = 4), InterSense InertiaCube3	Unilateral: Sternum, humerus, scapula, FA (near wrist) adhesive tape and bandage	Sh ROM (HT and ST joint angles)	–	180° sh AB and EXT with wri in neutral position and elb extended	HS (*n* = 11) 8 M, 3 F 24.7 ± 4.2 Y	Analyse UL angular mobility and linear acceleration in three anatomical axes
Van Den Noort 2015, [36] Full-Text	M-IMU (*n* = 4), Xsens MTw	Unilateral: Thorax, Scapula, UA, FA (ISEO protocol [33, 37]) Straps, skin tape	Sh ROM (ST joint angles)	–	Humeral FLX (sagittal plane) and AB (frontal plane) with elb extended and thumb pointing up	P (*n* = 10) 8 M, 2 F 24–63 Y SD	Evaluate the change in 3D scapular kinematics using a single and double anatomical calibration with a scapular locator versus standard calibration; evaluate difference in 3D scapular kinematics between static posture and dynamic humeral elevation
Roldán-Jiménez e Cuesta-Vargas 2016, [38] Full-Text	M-IMU (*n* = 3), InterSense, InertiaCube3	Unilateral: Humerus (middle third, slightly posterior), scapula, sternum Double-sided tape, elastic bandage	Sh ROM (GH and ST joint angles)	–	Sh AB (frontal plane) Sh FLX (sagittal plane) Wri in neutral position and elb extended	Young HS (*n* = 11), 18–35 Y 3 M, 8 F Adult HS (*n* = 14) > 40 Y 5 M, 9F	Analyze differences in shoulder kinematics in terms of angular mobility and linear acceleration related to age
Wang 2017, [26] Full-Text	M-IMU (*n* = 3), Adafruit FLORA 9-DOF	Unilateral: Shoulder (flat part of acromion), spine (C7-T1, T4-T5) Zipped vest, elastic strap with Velcro	Sh ROM (ST joint angles), RMSE ~ 3.57°	PST-55/110 series	60 ° sh FLX with elb extended and thumb pointing up, place a cooking pot on a shelf, 40 ° sh EL in the scapular plane with elb extended and thumb pointing up, place a bottle of water on a shelf	P with musculoskeletal shoulder pain (*n* = 8) 3 M, 5 F 50 ± 6.44Y Physiotherapist (*n* = 5)	Evaluate usability of a smart garment-supported postural feedback scapular training in patients with musculoskeletal shoulder pain and in physiotherapists who take care patients with shoulder disorders
Aslani 2018, [39] Full-Text	M-IMU (*n* = 1), Bosh Sensortec BNO055 EMG, MyoWare Muscle Sensor	Unilateral: UA, deltoid (2 surface electrodes on each deltoid section) Band	Sh ROM (azimuthal and elevation angles)	–	Arm EL (medially, anteriorly, cranially, posteriorly, laterally) with elb fully extended	HS (*n* = 6) 4 M, 2 F 27.3 ± 3.4Y P (*n* = 1), M Frozen Sh 42 Y	Evaluate a measurements protocol to assess the performance of the shoulder by combining both ROM and electromyography measurements
Carbonaro 2018, [40] Conference	M-IMU (*n* = 3), Xsens MTw	Unilateral: Sternum, FA (distal), scapula (similar to the configuration in [37]) Elastic bands	Sh ROM (GH and ST joint angles)	–	Extra-rotation, Arm AB (with different load)	HS (*n* = 5)	Test a digital application intended for tele-monitoring and tele-rehabilitation of the shoulder muscular-skeletal diseases
Hurd 2018, [41] Full-Text	Acc buil.in ActiGraph (*n* = 2), ActiGraph GT3XP-BLE	Unilateral: Wrist, UA (mid-biceps level) Velcro strap	Sh ROM	–	ADL	P (*n* = 14) 7 M, 7 F 73 ± 6Y GH OA, RC disease	Evaluate changes in pain, self-reported function and objective measurement of upper limb activity after RSA
Langohr 2018, [6] Full-Text	IMU (*n* = 5), YEI Technology	Bilateral: Sternum, UA (lateral aspects of the midhumerus), FA (dorsal aspect of the wrist) Shirt	Sh ROM (humeral elevation and plane of elevation angles)	–	Daily activities (1 day, monitoring of 11 ± 3 h)	P (*n* = 36) 73 ± 10 Y TSA, RTSA	Determine the total daily shoulder motion of patients after TSA and RTSA, compare the motion of the arthroplasty shoulder with that of the contralateral asymptomatic joint and compare the daily motion of TSA and RTSA shoulders

*acc* accelerometer, *gyr* gyroscope, *magn* magnetometer, *IMU* Inertial Measurement Unit, *M-IMU* Magneto and Inertial Measurement Unit, *UA* Upper Arm, *FA* Forearm, *ROM* Range of motion, *HT* humerothoracic, *ST* scapulothoracic, *GH* glenohumeral, *Sh* shoulder*, wri* wrist, *elb* elbow, *FLX-EXT* flexion-extension, *AB-AD* abduction-adduction, *IER* internal-external rotation, *RMSE* root mean square error, r = correlation, *r*^2^, coefficient of determination, *HS* Healthy subject, *CG* Control Group, *P* patient, *M* male, *F* female, *RC* Rotator Cuff, *OA* Osteoarthritis, *Y* Years old, *SST* Simple Shoulder test, *DASH* Disability of the Arm, Shoulder and Hand, *CMS* Constant Murley Score

**Table 2:** Shoulder motion monitoring for application in patients with neurological disorders

Reference, Year, Type of publication	Sensors, Brand	Placement and wearability	Target shoulder parameters, Performance	Gold standard	Task executed	Participants	Aim
Bartalesi 2005, [8] Conference	Strain sensor, WACKER Ltd. (ELASTOSIL LR 3162 A/B)	Unilateral: Sensors’ segment series along right upper limb Shirt	Sh ROM	–	–	HS	Wearable garment able to reconstruct shoulder, wrist and elbow movement to correct stroke patients’ rehabilitation exercises
Hester 2006, [42] Conference	Acc (*n* = 6)	Unilateral: thumb, index, back of the hand, FA and UA (medially), thorax Adhesive	Sh ROM	–	Reaching, prehension, manipulation	SP (*n* = 12)	Predict clinical scores of stroke patients’ motor abilities
Zhou 2006, [43] Full-Text	IMU (*n* = 2), Xsens MT9B	Unilateral: Wrist (inwards), elbow (outwards) -	Sh orientation and position	–	FA FLX-EXT FA PR-SU, reach test	HS (n = 1, M)	Propose a data fusion algorithm to locate the shoulder joint without drift
Willmann 2007, [44] Conference	M-IMU (*n* = 4), Philips	Unilateral: Torso, shoulder, UA, FA Garment	Sh ROM	–	–	HS	Provide a home system for upper limb rehabilitation in stroke patients
Zhou 2008, [45] Full-Text	M-IMU (*n* = 2), Xsens MT9B	Unilateral: FA (distally, near the wrist center), UA (laterally, on the line between the lateral epicondyle and the acromion process) Velcro straps	Sh orientation and position, RMSE = 2.5°-4.8°	CODA	reaching shrugging FA rotation	HS (*n* = 4, M) 20–40 Y	Validate data fusion algorithm
Giorgino 2009, [46] Full-Text	Strain sensor (*n* = 19), WACKER Ltd. (ELASTOSIL LR 3162 A/B)	Unilateral: Sensors’ sensing segments distributed over the UA, FA, shoulder, elbow, wrist Shirt	Sh ROM	–	GH FLX (sagittal plane) Lateral AB ER	HS (*n* = 1)	Describe a sensing garment for posture recognition in neurological rehabilitation
Lee 2010, [47] Full-Text	Acc (*n* = 2), Freescale MMA7261 QT	Unilateral: UA, FA Velcro strap	Sh ROM, Mean error~0°-3.5°	gon	FLX-EXT (sagittal plane)	HS (*n* = 1)	Validate performance and accuracy of the system
Chee Kian 2010, [48] Conference	OLE (*n* = 1) Acc (*n* = 1)	Unilateral: UA, elbow Adhesive patch	Sh ROM	IGS-190	Cyclic movements with arm exerciser	HS (*n* = 1)	Validate feasibility and performance (accuracy, repeatability) of the proposed sensing system designed to assist stroke patients in upper limb home rehabilitation
Patel 2010, [49] Full-Text	Acc (*n* = 6)	Unilateral: thumb, index, back of the hand, FA, UA, trunk -	Sh ROM	–	8 activities of FAS	SP (*n* = 24) 57.5 ± 11.8 Y	Evaluate accuracy of FAS score obtained via analysis of the accelerometers data comparing such estimates with scores provided by an expert clinician using this scale
Pérez 2010, [15] Full-Text	M-IMU (*n* = 4), Xsens MTi	Unilateral: UA (18 cm from acromion), FA (25 cm from epicondyle), hand (5.5 cm from distal radio-cubital joint), back (position is not relevant) Strap	Sh ROM, *r* = 0.997 (for sh IR, after calibration)	BTS SMART-D	Sh FLX-EXT Sh horizontal AB-AD Sh IR Elb FLX Elb PR-SU Wri FLX-EXT Serving water from a jar	HS (*n* = 1, F)	Validate the system
Bento 2011, [50] Conference	M-IMU (*n* = 4)	Bilateral: Shoulder, UA, wrist (affected and unaffected side) Strap	Sh orientation and position	–	FA to Table FA to box Extend elbow Hand to table Hand to box	SP (*n* = 5, M) 35–73 Y	Preliminary validation of a system able to quantify upper limb motor function in patients after neurological trauma
Nguyen 2011, [51] Full-Text	OLE (*n* = 3) Acc (*n* = 3)	Unilateral: Shoulder, elbow, wrist Clothing module fixed with Velcro straps	Sh ROM, Test2: RMSE = 3.8° (gon), RMSE = 3.1° (SW) Test3: ICC = 0.975 (sh)	Test2: gon, Shape- Wrap	Test2: Bend and Flex elbow Test3: reaching task	Test2: HS (n = 3, M); Test3: HS (*n* = 5)	Validation of the proposed motion capture system
Ding 2013, [52] Full-Text	M-IMU (*n* = 2), Analog Device (acc: ADXL320), HoneyWell (magn: HMC1 053), Silicon Sensing System (gyr)	Unilateral: UA (distal, near elbow), FA (distal, near wrist) Velcro strap	Sh ROM	–	Replication of 10 reference arm posture	HS (*n* = 5) 20–27 Y	Check the feasibility of the proposed system which measures orientation and corrects upper limb posture using vibrotactile actuators
Lee 2014, [53] Full-Text	M-IMU (*n* = 7), Analog Device (acc:ADXL 345) InvenSense (gyr: ITG3200) Honeywell (magn: HMC5883L)	Bilateral: Back, UA, FA and hand Strap	Sh ROM, Test1: r = 0.963 Test2: RMSE< 5°	Test1: gon Test2: VICON	Sh FLX-EXT Sh AB Arm IER Elb FLX FA PR-SU Wri FLX-EXT Wri radial-ulnar deviation	SP (*n* = 5) 2 M, 3 F Mean age: 68 Y	Introduce a smartphone centric wireless wearable system able to automate joint ROM measurements and detect the type of activities in stroke patients
Bai 2015, [54] Full-Text	M-IMU (*n* = 4 or *n* = 1), Xsens MTx	Unilateral Scapula, UA, FA, back of the hand or only on UA Velfoam, Velcro straps	Sh ROM (upper limb segments orientation and position)	Test1: gon Test2: gon, VICON	Sh FLX-EXT Sh IER Sh AB-AD Elb FLX-EXT FA PR-SU Wri FLX-EXT Wri radial-ulnar deviation	HS (*n* = 10) 8 M, 2 F 20–38 Y P (n = 1), F 41 Y	Evaluate a four-sensor system and a one-sensor system, investigate whether these systems are able to obtain quantitative motion information from patients’ assessment during neurorehabilitation
Ertzgaard 2016, [55] Full-Text	IMU (*n* = 5), Analog Device, Adis 16,350	Bilateral: Back (upper body), UA and FA Strap	Sh ROM (HT joint angles), ICC = 0.768–0.985	Coda	Movements that mimic activities of daily life	HS (*n* = 10) 2 M, 8 F 34.3 ± 13.1Y SP (*n* = 1), F, 43 Y	Validation study to characterize elbow and shoulder motion during functional task using a modified Exposure Variation Analysis (EVA)
Lorussi 2016, [56] Full-Text	M-IMU (*n* = 2), Xsens MTw Strain sensor (*n* = 1), Smartex	Unilateral: M-IMU sensors on sternum and UA Textile-based strain sensor on the back (from the spine to scapula) Shirt	Sh ROM (HT joint angle and scapular translation)	BTS SMART-DX	UA FLX (sagittal plane) UA AB (frontal plane)	HS (*n* = 5)	Validate sensors and data fusion algorithm to reconstruct scapular-humeral rhythm
Mazamenos 2016, [57] Full-Text	M-IMU (*n* = 2), Shimmer2r	Unilateral: UA (distal, near elbow), FA (distal, near wrist) Straps	Sh ROM (segments’ orientation and position)	–	EXP1: Reach and retrieve, lift object to mouth, rotate an object EXP2: preparing a cup of tea	HS (*n* = 18) M, F 25–50 Y SP (n = 4) M, F 45–73 Y	Evaluate the performance and robustness of a detection and discrimination algorithm of arm movements
Jiang 2017, [58] Conference	Acc (*n* = 4), Analog Device ADXL362 EMG (n = 4), Analog Device AD8232 Temperature (*n* = 1)	Unilateral: along upper limb Shirt	Sh ROM	–	4 typical joint actions performed in clinical assessment	HS (*n* = 1), M	Introduce an IoT-Bases upper limb rehabilitation assessment system for stroke patients
Li 2017, [59] Full-Text	IMU (*n* = 2), InvenSense, MPU-9250 EMG (n = 10), American Imex, Dermatrode	Unilateral: IMU: UA, wrist EMG: FA (*n* = 8), UA (*n* = 2) Stretchable belt	Sh ROM	–	11 tasks including: Sh FLX, Sh AB Wri FLX-EXT, Fetch and hold a ball or a cylindric roll, finger to nose, touch the back of the sh, FA PR-SU	HS (*n* = 16) 10 M, 6 F 36.25 ± 15.19Y SP (*n* = 18) 11 M, 7 F 55.28 ± 12.25 Y	Propose data fusion from IMU and surface EMG for quantitative motor function evaluation in stroke subjects
Newman 2017, [60] Full-Text	IMU (n = 3), Gait Up SA Physilog4	Bilateral: sternum, UA (posterior) Velcro straps, adhesive patch	Sh ROM (HT joint angles)	–	Reaching movements (lateral, forward, upward)	Children (*n* = 30) 10.6 ± 3.4 Y 17 boys, 13 girls	Test an IMU-based system to measure upper limb function in children with hemiparesis and its correlation with clinical scores
Yang e Tan 2017, [61] Conference	M-IMU (*n* = 4), APDM Opal	Unilateral: Waist, thorax, UA (distal, near elbow), FA (distal, near elbow) Straps	Sh ROM (HT joint angles)	Optitrack	Movements related with waist joint, sh joint and elb joints to achieve joint rotation	HS (*n* = 2)	Validate the proposed motion tracking system
Daunoraviciene 2018, [62] Full-Text	M-IMU (*n* = 6), Shimmer	Bilateral: UA, FA, hand (on centres of mass) Strap	Sh ROM	–	FNT test (Sh EXT, sh AB, elb FLX, hand SU)	CG (*n* = 24) 7 M 31.14 ± 5.67 17 F 28 ± 3.97 MS-P (*n* = 34) 13 M 36.46 ± 13.07 21 F 42.19 ± 12.55	Test a M-IMU based system to identify quantitative parameters for evaluation of UL disability and relate it to clinical scores
Jung 2018, [63] Conference	M-IMU (*n* = 5), Xsens MTw Awinda	Bilateral: UA and FA, trunk Velcro straps	Sh ROM (HT join angles), RMSE = 0.32 for the estimation of movements qualities using data of the entire duration of movements	Quality of movements’ label provided by the therapist	Reaching exercise	SP (*n* = 5), F 66.6 ± 15.9 Y	Evaluate movements quality regarding compensation and inter-joint coordination; exploiting a supervised machine learning approach, validate the hypothesis that therapists’ evaluation can be made considering only the beginning movement data
Lin 2018, [64] Full-Text	IMU (*n* = 2)	Unilateral: UA (distal, near elbow), FA (dorsal aspect of the wrist) Strap	Sh ROM	–	Sh FLX-EXT Sh AB Sh ER Elb FLX FA PR-SU	SP (*n* = 18): *n* = 9, control group (62.6 ± 7.1) n = 9, device group (52.2 ± 10.2 Y)	Evaluate the feasibility and efficacy of an IMU-based system for upper limb rehabilitation in stroke patients and compare the intervention effects with those in a control group
Repnik 2018, [7] Full-Text	M-IMU (*n* = 7), Myo armband, *n* = 2_ with *n* = 8 EMGs built-in, Thalamic labs	Bilateral: -M-IMU: Back of the hand, wrist, UA (distal, near elbow), sternum -MYO: FA (in the proximity of elbow joint) Straps, armband	Sh ROM (HT joint angles)	–	ARAT tasks: 19 movements divided in 4 subtests (grasp, grip, pinch, gross arm movement)	SP (*n* = 28) 18 M, 10 F 57 ± 9.1 Y HS (*n* = 12) 9 M, 3 F 36 ± 8 Y	Quantify UL and trunk movement in stroke patients

*acc* accelerometer, *gyr* gyroscope, *magn* magnetometer, *OLE* Optical Linear Encoder, *IMU* Inertial Measurement Unit, *M-IMU* Magneto and Inertial Measurement Unit, *UA* Upper Arm, *FA* Forearm, *ROM* Range of motion, *HT* humerothoracic, *GH* glenohumeral, *Sh* shoulder*, wri* wrist, *elb* elbow, *FLX-EXT* flexion-extension, *PR-SU* pronation-supination, *AB-AD* abduction-adduction, *IER* internal-external rotation, *RMSE* root mean square error, r = correlation, *ICC* Intraclass Correlation Coefficients, *gon* goniometer, *HS* Healthy subject, *CG* control group, *SP* Stroke Patient, *MS-P* Multiple Sclerosis Patient, *P* patient, *M* male, *F* female, *Y* Years old, *FAS* Functional Ability Scale, *ARAT* Action research arm test

**Table 3:** Shoulder motion monitoring for application in patients undergoing rehabilitation

Reference, Year, Type of publication	Sensors, Brand	Placement and wearability	Target shoulder parameters, Performance	Gold standard	Task executed	Participants	Aim
Cutti 2008, [37] Full-Text	M-IMU (*n* = 4), Xsens MT9B	Unilateral: Thorax (flat portion of the sternum), Scapula (central third, aligned with the cranial edge of the scapular spine), humerus (central third, slightly posterior), FA (distal) Double-sided tape, elastic cuff	Sh ROM (HT and ST joint angles) RMSE = 0.2°-3.2°	VICON	Exp 1: elb FLX-EXT, PR-SU sh FLX-EXT, IER, sh EL-DE and P-R Exp 2: tasks in Exp1 + sh IER (arm abducted 90°), sh AB-AD (frontal plane), HTN (sagittal and frontal plane)	HS (*n* = 1, M)	Develop a protocol to measure humerothoracic, scapulothoracic and elbow kinematics
Parel 2012, [65] Full-Text	M-IMU (*n* = 3), Xsens MTx	Unilateral: thorax, scapula, humerus Elastic cuff, adhesive	Sh ROM (HT and ST joint angles)	–	Humerus FLX-EXT (sagittal plane) and AB-AD (scapular plane)	HS (*n* = 20) 7F, 13 M 28.3 ± 5.5 Y P (*n* = 20) 8F, 12 M 43.9 ± 19.9 Y	Assess the inta- and inter-operator agreement of ISEO protocol in measuring scapulohumeral rhythm
Daponte 2013a, [66] Conference	M-IMU (*n* = 2), Zolertia Z1	Unilateral: UA, FA Brace	Sh ROM	–	Sh AB-AD Elb FLX	HS (*n* = 1)	Discuss design and implementation of a home rehabilitation system
Daponte 2013b, [67] Conference	M-IMU (*n* = 2), Zolertia Z1	Unilateral: UA, FA Brace	Sh ROM, Test1: max gap = 6.5° (roll) 5.2° (pitch) 11.6° (yaw)	Test 1: Tecno Body MJS Test 2: BTS	Test 1: shoulder IR, EL-DE and horizontal FLX-EXT Test 2: elbow FLX-EXT (along sagittal and horizontal plane)	HS (*n* = 1, M)	Validation of a home rehabilitation system
Pan 2013, [68] Full-Text	Acc (*n* = 2), LIS3LV02DQ Acc built-in a Smartphone (*n* = 1)	Unilateral: -acc: UA, thorax -Smartphone: wrist Strap, Armband	Sh ROM (HT joint angles)	–	Touch ear Use fingers to climb wall Pendulum clockwise and counter clockwise Active-assisted stretch fore and side, raises hand from back	HS (*n* = 10) 3 M, 7 F 20-25Y P (n = 14) 5 M, 9 F 44–67 Y	Describe design and implementation of a shoulder joint home-based rehabilitation monitoring system
Thiemjarus 2013, [69] Conference	Acc (*n* = 1), Analog Device (acc: ADXL330) Magn (*n* = 1), Honeywell (magn: HMC5843)	Unilateral: UA (proximal) or wrist, left or right Strap	Sh ROM, RMSE = 0.86°-5.05°	–	Sh FLX-EXT, AB-AD, horizontal AB-AD, IER	HS, (*n* = 23) 20–55 Y	Evaluate the effect of sensor placement on the estimation accuracy of shoulder ROM
Rawashdeh 2015, [70] Conference	M-IMU (*n* = 1), InvenSense (gyr: ITG-3200) Analog Device (acc: ADXL 345) Honeywell (magn: HMC5883L)	Unilateral: UA (lateral) Strap	Sh ROM	–	7 sh rehabilitation exercises 2 sports activities	HS (*n* = 11)	Describe a detection and classification method of shoulder motion gestures that can be used to prevent shoulder injury
Álvarez 2016, [71] Full-Text	M-IMU (*n* = 4), Xsens MTx	Unilateral: Back of the hand, FA (near wrist), UA (near elbow), back Wristband, Velcro strap, elastic band	Sh ROM, Lab test: mean error = 0.06° (FLX) 1.05° (lateral deviation)	Lab test: robotic wrist	Test1: Mounting of a shock dumper system Test2: holding a tablet for long periods Test3: elbow FLX-EXT	Test1: Mechanical worker (*n* = 1) Test2: worker of a commercial centre (*n* = 1) Test3: patient (*n* = 1)	Demonstrate the feasibility of an IMU-based system to measure upper limb joint angles in occupational health
Lee 2016, [72] Conference	Strain sensor (*n* = 2), MWCNT, Hyosung: multi-walled carbon nanotubes, EcoFlex0030, Smooth-On: silicon rubber	Unilateral: Shoulder Skin adhesive	Sh ROM, RMSE<10°	OptiTrack	Sh FLX-EXT Sh AB-AD	HS (*n* = 1)	Validate sensors and calibration method estimating two shoulder joint angles
Tran e Vajerano 2016, [73] Conference	M-IMU (*n* = 2), Shimmer2r	Unilateral: UA (distal, near elbow), FA (distal, near wrist) Straps	Sh ROM (HT joint angles)	–	Periodic arm movements	HS (*n* = 1)	Validate an algorithm to predict the received signal strength indicator (RSSI) and the future joint-angle values of the user
Rawashdeh 2016, [74] Full-Text	M-IMU (*n* = 1), InvenSense (gyr: ITG-3200) Analog Device (acc: ADXL 345) Honeywell (magn: HMC5883L)	Unilateral: UA (central third) Straps	Sh ROM (HT motion)	Visual observation	7 sh rehabilitation exercises, baseball throws, volley serves	HS (*n* = 11) 25 ± 7 Y	Validate a detection and classification algorithm of upper limb movements
Wu 2016, [75] Full-Text	M-IMU (*n* = 3), Bluetooth 3-Space Sensor, YEI	Unilateral: FA (near wrist), UA (near elbow), thorax (shifted to the right) Strap, bandage	Sh ROM (HT joint angles)	–	12 gestures common in daily life: 3 static and 9 dynamic	HS (*n* = 10) 9 M, 1 F 22–24 Y	Evaluate accuracy, recall and precision of a gesture recognition system
Burns 2018, [27] Full-Text	Acc and gyr built-in a smartwatch (*n* = 1), Apple Watch (Series 2 &3)	Unilateral: Wrist Wristband	Sh ROM	–	Pendulum AB Forward EL IR ER Trapezius EXT Upright row	HS (*n* = 20) 14 M, 6 F 19–56 Y	Evaluate performance of a commercial smartwatch to perform home shoulder physiotherapy monitoring
Esfahani e Nussbaum 2018, [11] Full-Text	Textile sensors (printed) *n* = 11	Bilateral: Shoulder (*n* = 6), low back (*n* = 5) Undershirt	Sh ROM, mean error = 9.6° for sh angle estimation	M-IMU (Xsens MTw Awinda)	Sh AB Sh FLX-EXT Sh IER Left/right side bending Trunk FLX-EXT Trunk rot left/right	HS (*n* = 16), 10 M, 6 F 21.9 ± 3.3	Describe a smart undershirt and evaluate its accuracy in task classification and planar angle measures in the shoulder joints and low back
Ramkumar 2018, [76] Full-Text	Acc,gyr and magn built-in a smartphone, Apple iPhone	Unilateral: UA, FA Armband	Sh ROM <5°	gon	AB (coronal plane) forward FLX (sagittal plane) IER (elbow fixed to the body flexed to 90°)	HS (*n* = 10) 5 M, 5 F Mean 27 Y	Validate a motion-based machine learning software development kit for shoulder ROM

*acc* accelerometer, *gyr* gyroscope, *magn* magnetometer, *IMU* Inertial Measurement Unit, *M-IMU* Magneto and Inertial Measurement Unit, *UA* Upper Arm, *FA* Forearm, *ROM* Range of motion, *HT* humerothoracic, *ST* scapulothoracic, *Sh* shoulder*, elb* elbow, *FLX-EXT* flexion-extension, *AB-AD* abduction-adduction, *IER* internal-external rotation, P-R protraction-retraction, *RMSE* root mean square error, *HS* Healthy subject, *P* patient, *M* male, *F* female, *Y* Years old

**Table 4:** Studies focused on validation/development of systems/algorithms for monitoring shoulder motion

Reference, Year, Type of publication	Sensors, Brand	Placement and wearability	Target shoulder parameters, Performance	Gold standard	Task executed	Participants	Aim
Jung 2010, [77] Conference	IMU (*n* = 6), ADXL 345 (acc) LPY51 50 AL (gyr) HMC5843 (magn)	Bilateral: UA and FA (distal third), thorax and pelvis Strap	Sh orientation and position	HDRT system	Arms above head Bend arms Bend waist	HS (*n* = 1)	Validate the motion tracking algorithm
El-Gohary 2011, [78] Conference	IMU (*n* = 2), APDM Opal	Unilateral: FA (near wrist), UA (distal third) Strap	Sh ROM, r = 0.91–0.97	Eagle Analog System	Sh FLX-EXT Sh AB-AD Elb FLX-EXT Elb PR-SU	HS (*n* = 1)	Validate data fusion algorithm
Zhang 2011, [79] Full-Text	M-IMU (*n* = 3), Xsens MTx	Unilateral: UA (laterally, above the elbow), FA (lateral and flat side of the FA near the wrist), sternum Strap, clothing	Sh ROM, RMSE = 2.4° (sh FLX-EXT) RMSE = 0.9° (sh AB-AD) RMSE = 2.9° (sh IER)	BTS SMART-D	Free movements	HS (*n* = 4)	Validate sensor fusion algorithm
El-Gohary 2012, [80] Full-Text	IMU (*n* = 2), APDM Opal	Unilateral: UA (middle third, slightly posterior), FA (distal, near wrist) Strap band	Sh ROM, RMSE = 5.5° (sh FLX-EXT) RMSE = 4.4° (sh AB-AD)	VICON	Sh FLX-EXT Sh AB-AD Elb FLX-EXT FA PR-SU Touching nose Reaching for a doorknob	HS (n = 8)	Validate data fusion algorithm
Lee e Low 2012, [81] Full-Text	Acc (n = 2), Freescale MMA7361 L	Unilateral: UA (near elbow), FA (near wrist) -	Sh ROM, RMSE = 2.12° (sh FLX-EXT) RMSE = 3.68° (sh rotation)	IMU (Xsens MTx)	UA FLX-EXT and medial/lateral rotation FA FLX-EXT and PR-SU (sagittal plane)	HS (*n* = 1)	Validate the feasibility of the proposed algorithm
Hsu 2013, [82] Conference	M-IMU (*n* = 2), LSM303DLH (acc, magn) L3G4200D (gyr)	Unilateral: UA, FA Velcro strap	Sh ROM, RMSE = 1.34°-5.08°	Xsens MTw, (*n* = 2)	Sh FLX, AB, EXT, ER and IR	HS (n = 10) 8 M, 2 F 23.3 ± 1.33 Y	Validate data fusion algorithm
Lambrecht e Kirsch 2014, [16] Full-Text	M-IMU (n = 4), InvenSense MPU-9150 chip	Unilateral: Sternum, UA, FA and hand -	Sh ROM, RMSE = 4.9° (sh azimuth) RMSE = 1.2° (sh elevation) RMSE = 2.9° (sh IR)	Optotrack	Reaching movements	HS (*n* = 1)	Validate sensors’ accuracy and data fusion algorithm
Ricci 2014, [83] Full-Text	M-IMU (n = 5), APDM Opal	Bilateral: Thorax, UA (latero-distally) and FA (near wrist) Velcro strap	Sh ROM (HT joint angles)	–	UA FLX-EXT UA AB-AD FA PR-SU FA FLX-EXT Thorax rotation Thorax FLX-EXT	Children (*n* = 40) 6.9 ± 0.65 Y	Develop a calibration protocol for Thorax and upper limb motion capture
Roldan-Jimenez 2015, [84] Full-Text	Inertial sensors built-in a Smartphone (*n* = 1), LG Electronics INC, iPhone4	Unilateral: UA Neoprene arm belt	Sh ROM	–	sh AB, EXT with wrist in neutral position and elb extended	HS (n = 10) 7 M, 3 F 24.2 ± 4.04 Y	Study humerus kinematics through six physical properties that correspond to angular mobility and acceleration in the three axes of space
Fantozzi 2016, [17] Full-Text	M-IMU (*n* = 7), APDM Opal	Bilateral: Sternum, UA, FA, back of the hand Velcro strap	Sh ROM (HT joint angles), RMSE <10° (sh FLX-EXT, AB-AD, IER)	BTS SMART-DX	Simulated front-crawl and breaststroke swimming	HS (n = 8), M 26.1 ± 3.4 Y	Validate a protocol to assess the 3D joint kinematics of the upper limb during swimming
Meng 2016, [85] Full-Text	M-IMU (*n* = 2), Shimmer2r	Unilateral: UA (distal, near elbow), FA (distal, near wrist) Straps	Sh ROM, Test2: RMSE = from 2.20° to 0.87°	VICON	Sh FLX-EXT Sh AB-AD Sh IER Elb FLX-EXT Elb PR-SU	Test1: HS (n = 15), M, 19–23 Y Test2: HS (*n* = 5)	Validate an algorithm to improve accuracy on measurements of arm joint angles considering the properties of human tissue
Crabolu 2017, [86] Full-Text	M-IMU (n = 3), Xsens, MTw2 Awinda	Unilateral: UA, scapula, Sternum Velcro strap, double-sided tape, elastic band	GH joint center	MRI acquisition	Cross and star motions (2 joint velocities, 2 range of motions)	HS (*n* = 5) 3 M, 2 F 36 ± 4 Y	Evaluate accuracy and precision of the GHJC estimation
Kim 2017, [87] Conference	MYO armband (n = 1): contains 8 EMG and 1 IMU, Thalamic Labs, MYO armband	Unilateral: UA (near elbow) Armband	Sh ROM	–	Elb FLX (0°,45°,90°) with sh in neutral position, elb FLX (0°,45°,90°) with sh FLX 90° (sagittal plane)	HS (n = 1)	Introduce an algorithm for upper arm and forearm motion estimation using MYO armband
Morrow 2017, [88] Full-Text	M-IMU (n = 6), APDM Opal	Bilateral: FA and UA (lateral), head, sternum Velcro straps	Sh ROM (HT joint angles), RMSE = 6.8° ± 2.7° (sh elevation)	Raptor 12 Digital Real Time Motion Capture System	Peg transfer (to mimic minimally invasive laparoscopy)	Surgeon HS (n = 6) 3 M, 3 F 45 ± 7 Y	Validate a M-IMU based protocol to measure shoulder EL, elbow FLX, trunk FLX-EXT and neck FLX-EXT kinematics
Rose 2017, [89] Full-Text	IMU (*n* = 6), APDM Opal	Bilateral: UA (lateral), FA (dorsal), sternum, lumbar spine Straps	Sh ROM (HT joint angles)	–	Diagnostic arthroscopy simulation	Surgeon HS (*n* = 14)	Develop an IMU-based system to assess the performance of orthopaedic residents with different arthroscopic experiences
Tian 2017, [90] Conference	M-IMU (n = 2), Acc: LIS3LV02D, Magn: HMC5843, Gyr: ITG3200	Unilateral: UA, FA Straps	Sh ROM	VICON	Sh FLX and elb FLX (sagittal plane)	HS (*n* = 1)	Validate data fusion algorithm
Pathirana 2018, [91] Full-Text	M-IMU (*n* = 1)	Unilateral: Wrist Strap	Sh ROM	VICON, Kinect	Forward FLX-EXT AB-AD Backward FLX-EXT Horizontal FLX-EXT	HS (*n* = 14) 10 M, 4 F	Validate accuracy and robustness of data fusion algorithm using a single sensor to measure shoulder joint angles

*acc* accelerometer, *gyr* gyroscope, *magn* magnetometer, *IMU* Inertial Measurement Unit, *M-IMU* Magneto and Inertial Measurement Unit, *UA* Upper Arm, *FA* Forearm, *ROM* Range of motion, *HT* humerothoracic, *GH* glenohumeral, *Sh* shoulder*, elb* elbow, *FLX-EXT* flexion-extension, *PR-SU* pronation-supination, *AB-AD* abduction-adduction, *IER* internal-external rotation, *RMSE* root mean square error, r = correlation, *HS* Healthy subject, *M* male, *F* female, *Y* Years old

### Sensing technology

Some studies combined different sensors in their measurements system. The most used sensors are accelerometers, gyroscopes and magnetometers, a combination of them (*n* = 55) or with other sensors (*n* = 8), or built-in into other devices (e.g., smartphones, smartwatch) (*n* = 6); additional studies (*n* = 4) utilized strain sensors for motion analysis.

### B.1 wearable systems based on inertial sensors and magnetometers

An IMU allows estimating both translational and rotational movements. Such sensors comprise gyroscopes that measure angular velocity and accelerometers that measure proper acceleration, i.e. gravitational force (static) and force due to movements (dynamic) [92]. The main limitation of the gyroscopes is the issue bias due to drift. Gyroscopes do not have an external reference, as opposed to accelerometers that use gravity vector as reference; in the orientation estimation, gyroscopes suffer of drift during the integration procedures. To compensate such issue, these sensors are combined with magnetometers that measure magnetic field and use the Earth’s magnetic field as reference. The main limitation of magnetometers is the interference due to the presence of ferromagnetic materials in the surrounding environment [92]. We refer to these hybrid sensors as M-IMU (magnetic and inertial measurement unit). By integrating the information derived from each sensor (i.e., acceleration, angular velocity and magnetic field) through sensor-fusion algorithms, M-IMUs provide an accurate estimation of the 3D-position and 3D-orientation of a rigid body. The upper limb can be modelled as a kinematic chain constituted by a series of rigid segments, i.e., thorax, upper arm, forearm and hand, linked to each other by joints that allow relative motion among consecutive links [17]. In the kinematic chain, the shoulder joint consists of three degrees of freedom (DOFs) correspondent to abduction-adduction (AB-AD), internal-external rotation (IER), and flexion-extension (FLX-EXT) [15, 54, 57, 71, 79]. Shoulder rotations can be described using Euler angles that identify the anatomical DOFs with the roll-pitch-yaw angles [17, 33, 37, 88]. Sensor-fusion algorithms can exploit two main approaches, deterministic or stochastic. The deterministic approach includes the complementary filter that merges a high pass filter for gyroscope data (to avoid drift) and a low pass filter for accelerometer and magnetometer data [64, 82, 90, 92]. The stochastic approach includes the Kalman Filter and its more sophisticated versions [7, 55, 66, 67, 78–80, 91, 92]. The Kalman filter (KF) is the most used algorithm to process M-IMU and IMU data due to its accuracy and reliability [15, 38, 54, 75, 83, 93].

Wearable systems based on IMU or M-IMU include a variable number of sensor nodes that, properly distributed on each body segment of interest, provide kinematic parameters such as joint ROM, position, orientation, and velocity. Fifty-one out of the included studies used exclusively IMUs (*n* = 15) or M-IMUs (*n* = 36). Systems performances were analyzed in terms of the agreement between results obtained from the M-IMU or IMU-based systems and those collected by a gold standard system. Several types of systems were used as gold standard, such as ultrasound-based system (e.g., Zebris CMS-HS [29]), diagnostic imaging (e.g., Magnetic Resonance [86]), optical-based systems (e.g., VICON [37, 53, 54, 80, 85, 90, 91], BTS Bioengineering [15, 17, 56, 67, 79], Eagle Analogue System [78], Optotrack [16], Optitrack [61], CODA [45, 55]), goniometer [53, 54]. Results from an inertial system were benchmarked against an ultrasound-based reference system, showing a root mean square error (RMSE) of 5.81° and a mean error of 1.80° in the estimation of shoulder angles of FLX-EXT, AB-AD and IER evaluated in the sagittal, frontal and transversal planes, respectively [29]. Accuracy of a protocol based on commercial inertial sensors (MT9B, Xsens) was tested and compared to a VICON system to measure humerothoracic, scapulothoracic joint angles and elbow kinematics [37]. Results demonstrated high accuracy in the estimation of upper limb kinematics with an RMSE lower than 3.2° for 97% of data pairs. A BTS reference system was used to validate accuracy of a wearable system comprised of commercial sensors (Xsens) and results showed a mean error difference of 13.82° for FLX-EXT, 7.44° for AB-AD, 28.88° for IR [15]. In a protocol-validation study, commercial Opal sensors were compared to a BTS system to assess upper limb joint kinematics during simulated swimming movements. Data showed a median RMSE always better than 10° considering movements of AB-AD, IER and FLX-EXT in front-crawl and breaststroke [17]. Opal wearable sensors were compared to optical motion capture systems to estimate shoulder and elbow angles [78, 80]. Planar shoulder FLX-EXT and AB-AD were performed showing an RMSE of 5.5° and 4.4°, respectively [80]; a good correlation between the measurements performed on shoulder motion with the two systems was also found in [78] (no data regarding measurements error were proposed).

Some studies (*n* = 11) compared data obtained from wearable sensors, custom or commercial, with a gold standard to validate their own sensors data fusion algorithm (for more details see Table 4). Two different algorithms were compared to a customized KF [79]. Comparing the results derived from the BTS system and the inertial-based system (Xsens), the proposed algorithm showed a smaller error than the other two methods for computing shoulder FLX-EXT (RMSE = 2.4°), AB-AD (RMSE = 0.9°), IER (RMSE = 2.9°) [79]. The addition of the magnetometer-based heading correction in the sensor data fusion algorithm was investigated to test the accuracy of an inertial-based motion tracking system using the Optotrak Certus (Northern Digital Inc., Waterloo, ON, Canada) as reference. Results showed a RMSE of 4.9°, 1.2° and 2.9° for shoulder azimuth, elevation and internal rotation, respectively [16].

Four studies used only accelerometers [42, 47, 49, 81]. Systems performance analysis in measurement of arm motion, showed a RMSE lower than 3.5° and 3.68° for shoulder ROM when results from the accelerometers-based systems were benchmarked against a goniometer and commercial M-IMUs, respectively [47, 81]. Evaluation of upper limbs’ physical activity was performed recording data of accelerometers built-in wearable device as ActiGraph (Pensacola, Florida, Model GT3XP-BTLE) to obtain objective outcomes in patients after reverse shoulder arthroplasty [41].

Shoulder ROM has been also estimated by means of a single sensor node which integrated an accelerometer and a magnetometer [69]. Sensor fusion algorithms of accelerometers and magnetometers data provide accurate orientation estimation in static or semi static condition, e.g., in a rehabilitation session in which patients perform slow movements [81]. M-IMUs comprised of a 3D accelerometer, 3D gyroscope and 3D magnetometer are the most appropriate choice for motion tracking either in static that in dynamic condition.

Two accelerometer-based sensors were combined with those built-in a smartphone to realize a smart rehabilitation platform for shoulder home-rehabilitation [68]. Mobile phone or a smartwatch, with their built-in inertial sensor units, were used as mobile monitoring devices [27, 76, 84]. These results give proof of the growing trend in the application of commercial devices in clinical setting for rehabilitation purposes. Data has been processed using machine learning algorithms to extract salient features and for gesture recognition related to shoulder motion. In these techniques, the main steps are the data collection, followed by segmentation process, feature extraction and classification [27, 49]. For instance, the identification of different types of RC physiotherapy exercises has been performed processing data from inertial sensors built-in a wrist-worn smartwatch [27]. Data from inertial sensors built-in a smartphone were benchmarked against a manual goniometer. Angular differences between a machine learning-based application and goniometer measurements resulted less than 5° for all shoulder ROM (i.e., AD, forward FLX, IR, ER) [76].

Two studies combined accelerometer(s) with Optical Linear Encoder (OLE) [68, 84]. An OLE-based system acts as a goniometer providing measures of joint angles. Despite of the simplicity and low cost of the proposed systems, differences in shoulder ROM estimation resulted not negligible when data collected by the wearable systems were compared against an inertial-based motion capture (i.e., IGS-190 [54]) and a fiber optics-based system (i.e., ShapeWrap [71]).

Three studies included EMG sensors in their assessment tool in combination with accelerometers [58], IMUs [59] and M-IMU [39]. EMG sensors placed on the biceps, triceps [59] and deltoid muscles [39] provide additional information about upper limb motor function and shoulder assessment, evaluating muscles activity. Quantification of upper limb motion was executed through a wearable device, MYO armband by Thalamic labs, that combines EMG sensors to record electrical impulses of the muscles [7, 87].

### B.2 wearable systems based on strain sensors

Four studies used smart-textiles instrumented by strain sensors with piezoresistive properties to estimate kinematic parameters and to perform motion analysis [8, 11, 46, 56]. Such sensing elements are stretched or compressed during movements of the examined body segments, with consequent variation of their electrical resistance [94, 95]. Using a M-IMU system as reference, accuracy evaluation of a smart-textile with printed strain sensors showed a mean error of 9.6° in planar motions measurements of shoulder joint [11]. Shoulder kinematics was assessed combining a strain sensor for scapular sliding detection with two M-IMUs for HT orientation measure [56].

Piezoresistive strain sensors directly adhered to the skin were used to estimate shoulder ROM; the comparison between reference data from an optical-based system (i.e., Optitrack) and strain sensors showed a RMSE less than 10° in shoulder FLX-EXT and AB-AD estimation [72].

### Sensors placement and wearability

**Fig. 2:**
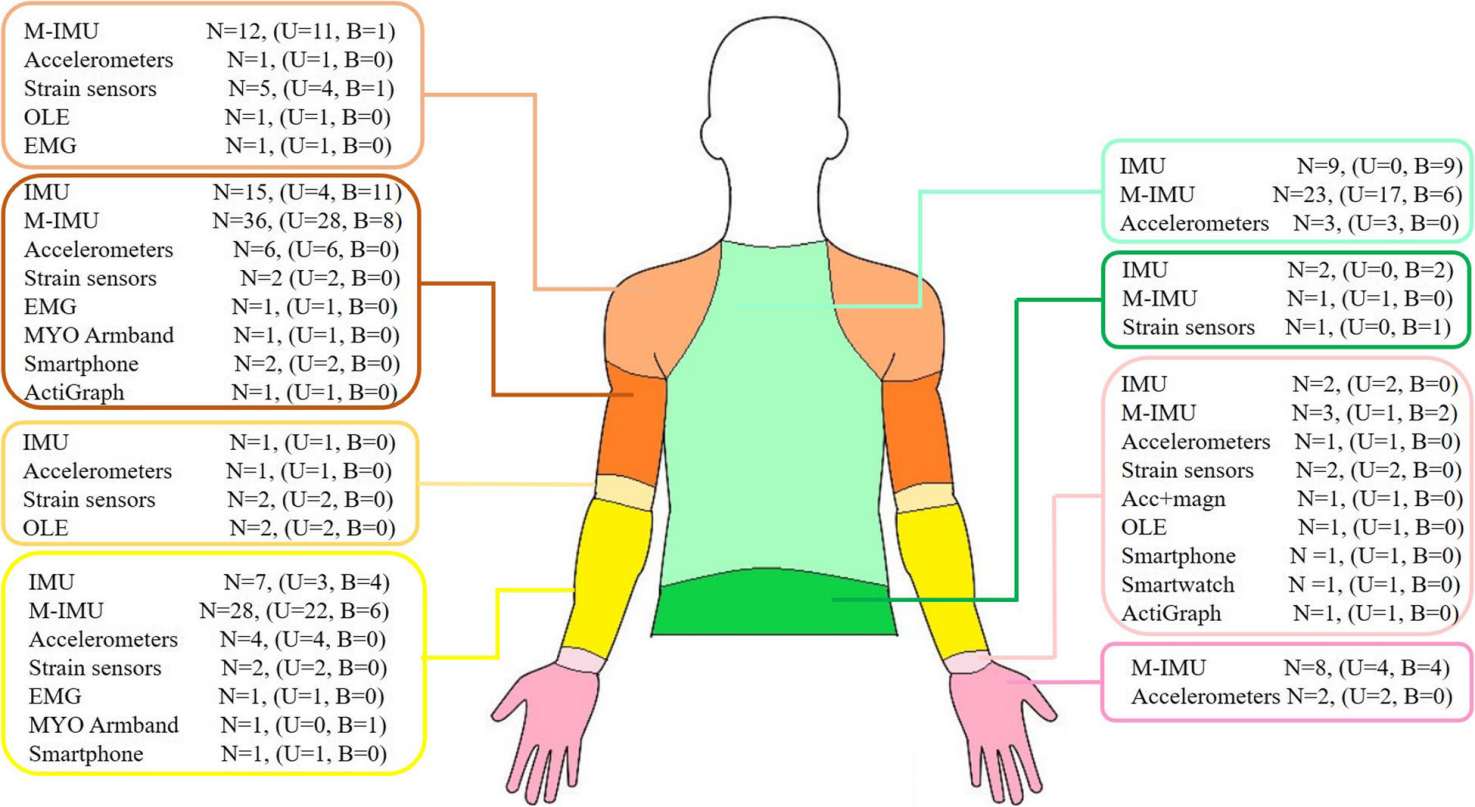
Placement of sensing units (NOTE One study [90] is not included because the specific position of each sensor nodes is not so clear. Legend: N = number of studies, U = Unilateral, B = Bilateral)

Placement of the sensing technology on the body landmarks has shown a heterogeneous distribution linked to the different nature of the employed technology and to the purpose for which monitoring system was designed. With respect to the monitored upper limb, 53 out of the 73 studies included in this review showed a unilateral distribution of the sensing elements while the remaining studies utilized a bilateral placement. Several configurations using different number of sensors and placements have been investigated as reported in detail in each table and Fig. 2.

Regarding the wearability, we classified the systems in terms of how the sensors were fixed to the human body: i) by adhesive patch, ii) by means of straps or embedded within pocket, iii) the sensing element is physically integrated into the fabric. Four studies did not specify the method of attachment, 12 studies have stuck sensors directly on human skin by means of adhesive patch, 52 studies have attached sensors through straps or embedding them in modular clothing, and 5 studies have integrated sensors directly into garments. For more details refers to Tables 1, 2, 3 and 4.

## Discussion

This paper summarizes the main features of wearable systems that have been employed in clinical setting and research field to evaluate upper limb functional performance and particularly for shoulder ROM assessment. Shoulder complex is characterized by the greatest mobility among all human joints and, due to its complexity, reviewed articles evidenced heterogeneity on the more suitable protocol for capturing joint ROM [96].

### Wearable technology

Although 73% of the reviewed papers use commercial products for tracking joint angles, many of these personalize the positioning of the sensors, the calibration methodology and the algorithms used to process the recorded data. This customization makes strenuous a direct comparison among protocols, especially if sensing units of different nature (e.g., M-IMU vs. strain sensors) are used to measure the same kinematic parameters, leaving still open the issue of the protocols’ definition with general validity.

About studies using inertial-based motion tracking systems, most in this summary (88%), calibration procedures before data acquisition and data processing represent a relevant issue about accuracy and reliability of the system. Typically, the M-IMUs are attached on the segment of interest to estimate its orientation, so the calibration is necessary to relate sensors’ measurements to movements of the tracked body segment. Sometimes the manufacturer suggests how to perform calibration, e.g., positioning sensors on a flat surface [15, 35] to align coordinate system or assuming static anatomical position [65], as N-pose [79], to compute orientation differences between segments and sensors coordinates in order to obtain sensor-to-segment alignment [56]. Dynamic or functional anatomical calibration has also been performed in some studies, but the sequence of movements executed varied among these [17, 33, 55, 83]. One interesting improvement that may be done to have a positive impact on the accuracy of inertial-based motion tracking systems, is to define a standard set of movements for the initial calibration and a standard method of data processing by which extrapolate kinematic parameters of high clinical relevance.

Some works have reported remarkable results in human motion tracking using e-textile sensors [8, 46]. Technological improvements in the development of conductive elastomers allowed to integrate such strain sensors directly into garments making them comfortable and unobtrusive [11, 56]. Although conductive elastomers ensure flexibility and performances comparable with those of the M-IMU sensors, the main limitations are the hysteresis, uniaxial measurements and non-negligible transient time [56]. Wearable systems based on strain sensors are a promising technology for kinematics analysis that may overcome the main M-IMUs drawbacks, as interferences due to surrounding ferromagnetic materials, gyroscopes’ error drift and long-term use. On the other hand, errors may occur with strain sensors-based systems in the estimation of shoulder kinematics for their inherent hysteresis behaviour.

Among wearable systems reviewed in this summary, differences resulted in terms of sensors typology, number and size, placement, and wearability features. Sensors placement and method of attachment must be carefully investigated as they could influence the outcomes reliability (e.g., effects of soft tissues’ artefacts). Human skeleton is covered by skin tissue and muscles. The combination of skin’s elasticity and muscle activity may cause negative effects in the measurement of the bones’ movement. In studies where M-IMU sensors were used to track shoulder kinematics, soft tissue properties were opportunely included in mathematical models to reduce soft tissue artifacts [79, 85]. The body fat percentage was found the main influencing factor that negatively affects the inertial sensors’ orientation [85]. To reduce such source of error, either when sensors are directly adherent to the skin that embedded in a textile, sensing units should be placed as near as possible to the bone segment to reduce soft tissue artifacts [97, 98]. Wearability is a key factor to consider because it can influence the level of patients’ acceptance [26].There are several relevant requirements that wearable systems must meet to encourage their applications in continuous monitoring of patient status. Indeed, execution of movements, either in home environments or in clinical settings, should not be hindered by measurements systems so they must be non-invasive, modular, lightweight, unobtrusive and include a minimal number of sensors [33, 40, 51, 56, 66, 67, 91]. Most studies have employed magneto and inertial-based tracking systems in which sensors were attached to the upper limb through Velcro straps or including them in modular brace and garments [26, 45, 67, 82, 88].

**Fig. 3:**
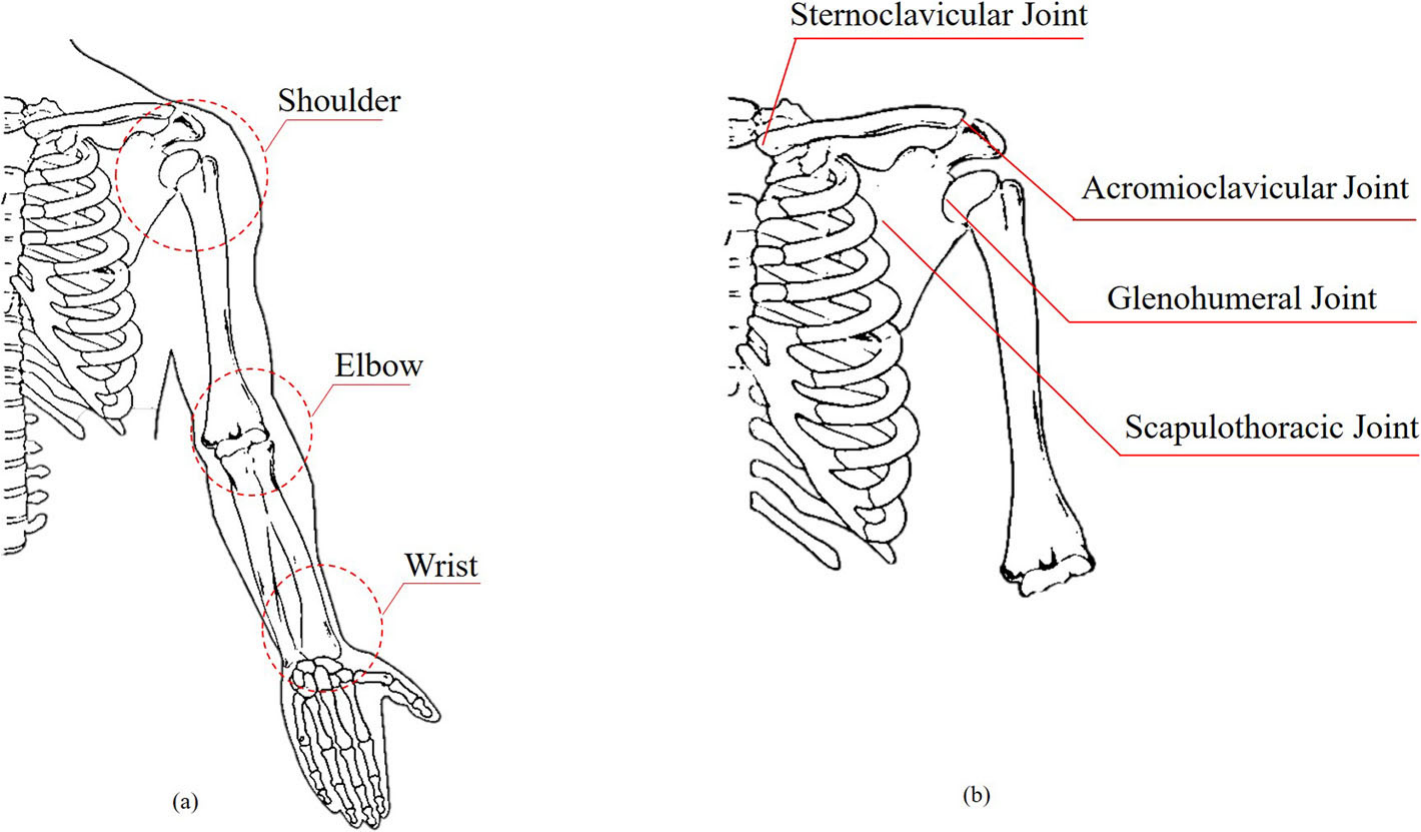
**a** Anatomy of the Upper limb; **b** Anatomy of the Shoulder complex

Upper limb includes the shoulder, elbow and wrist joints (Fig. 3a). Humerus, scapula, clavicle, and thorax constitute the shoulder complex: humeral head articulates in the glenoid fossa of the scapula to form GH joint, the AC joint is the articulation between the lateral end of the clavicle and the acromion process, the SC joint articulates the medial end of the clavicle and the sternum and the functional ST joint allows rotational and translational movements of the scapula with respect to the thorax [96] (Fig. 3b). The ST joint and GH joint act togheter in arm elevation according to scapular-humeral rhythm described in [99]. From a biomechanical point of view, shoulder complexity is justified by the high degree of coupling and coordination between shoulder joints (i.e.*,* shoulder rhythm) and the action of more than one muscles over more than one joints in the execution of a movement. Data extraction of shoulder kinematics is frequently based on movements pattern in the sagittal, frontal and transversal planes, so monitoring of complex movements (e.g., daily activities) in multiple planes, performed through wearable sensors, requires a more stringent evaluation and accurate interpretation. As resulted in the review, the shoulder is generally approximated as a ball-and-socket joint [56]. This assumption provides an approximate representation of the whole shoulder girdle (e.g., it neglects the contribution of scapular movements). A standardized protocol has been proposed (i.e., The ISEO®, INAIL Shoulder and Elbow Outpatient protocol) to improve the performance of M-IMUs in the estimation of scapular kinematics, by locating inertial sensors on the back in correspondence of scapula [33, 35–38, 40, 65]. An adequate investigation of scapular motions may be beneficial to assess shoulder disorders [100].

For long-term monitoring of shoulder kinematics considering also scapular motions, the combination of M-IMUs and smart-textile with embedded strain sensors is a perfect balancing of accuracy, flexibility and wearability (i.e., strain sensors positioned on the scapula could increase the portability and acceptance of the wearable system for long-term monitoring of ADLs) [86].

### Applicability in clinical setting and rehabilitation

Alterations in the complex shoulder kinematics can derive by both neurological or musculoskeletal disorders and result in pain and limited movements [68]. Compensatory movements in patients with shoulder disorders are the most common consequential responses to pain or to difficulty in performing free-pain movements. In such situations, information retrieved by posture monitoring may be beneficial in clinical application and rehabilitation [26]. In the last years, the application of wearable devices for gathering motion data outside the laboratory settings is growing. Avoiding complex laboratory set-up, wearable systems employed to assess upper limb kinematics have proven to be a well-founded alternative to obtain quantitative motions parameters. Quantitative outcomes about shoulder motions recorded by wearable sensors are beneficial in clinical practice in terms of time-saving and they are becoming a promising alternative to improve assessment accuracy overcoming the subjectivity of clinical scales. The automatic assessment of motor abilities can also provide therapists a tangible and, therefore, measurable awareness of the effectiveness of the treatment and the recovery path chosen.

In clinical practice, the severity level of patients’ condition with musculoskeletal disease is usually assessed through questionnaire-based scores [36, 42]. Algorithms for kinematic scores computing were developed to evaluate shoulder functional performance after surgery in subjects with GH osteoarthritis and RC diseases, elaborating data obtained from IMU sensors [29, 31]. High correlation (0.61–0.8) between shoulder kinematic scores (i.e., power score, range of angular velocity score and moment score) and clinical scales (e.g., DASH, SST, VAS) was found [31]. Unlike clinical scores, kinematic scores showed greater sensitivity in detecting significant functional changes in shoulder activity at each post-operative follow-up with respect to the baseline status [29, 31]. In a five-year follow-up study, asymmetry in shoulder movements was evaluated in patients with subacromial impingements syndrome. Asymmetry scores, derived from an IMU-based system, showed post-treatment improvements with greater sensitivity than clinical scores and only a weak correlation was found with DASH (r = 0.39) and SST (r = 0.32) [32]. Quantitative evaluation of arm usage and quality of movements in every kind of shoulder impairment contributes to outline a clinical picture about the functional recovery and the effectiveness of the treatment [30, 49]. Using the same number of IMU (*n* = 3) and the same placement on both humeri and sternum, the shoulder function was evaluated before and after treatment, in patients underwent surgery for RC tear [5, 34]. Results showed significative differences in movements frequency between patients and control group during activities of daily life [5], with limited use of arm at 3 months after surgery [34]. With a bilateral configuration based on 5 IMU, shoulder motion was assessed to extrapolate relevant clinical outcomes about Total Shoulder Arthroplasty (TSA) and Reverse Total Shoulder Arthroplasty (RTSA) [6]. Patients underwent either TSA or RTSA showed shoulder ROM below 80° of elevation, indiscriminately; but, on average, patients treated with RTSA performed movements above 100° less frequently [6]. Objective measurements (i.e., mean activity value and activity frequency) of limb function after RTSA did not show significant improvements 1 year after surgery, despite DASH scores and pain perception have improved compared to preoperative outcomes [41].

In patient with neurological impairments (e.g., stroke), assessments of motor abilities performed through wearable sensors showed a time saving compared to clinical scores (e.g., Fugl-Meyer Assessment Test) measured by the clinician [50, 53]. Data from accelerometers-based systems demonstrated accurate capability in the estimation of clinical scores for quality of movement (e.g., FAS score) and in prediction of shoulder features about shoulder portion of Fugl-Meyer scale with errors near 10% [42, 49]. Generally, the main evaluated features comprise coordination, smoothness, presence of compensatory movements, speed, amplitude of ROM. Quantitative measurements, such as movement time and smoothness, showed a strong correlation with Action research arm test scores in patients after stroke [7]. Spatiotemporal parameters (e.g., ROM, movement time) extracted from inertial sensors’ data provided an accurate evaluation of patients with multiple sclerosis and they distinguished affected and unaffected upper limbs in children with hemiparesis significantly [60, 62].

Digital simulations and virtual reality implementation in upper limb rehabilitation context aim to reproduce accurately limb movements processing data from wearable sensors and give a direct feedback about the adequacy or not of the executed movements [40]. The long-term monitoring, associated with suitable feedback strategy (e.g., visive, auditory, vibrational), can foster the correction of wrong postures [40, 52]. In addition, wearable systems allows a more supervised home-rehabilitation giving substantial improvements to patient healing: total patient involvement in rehabilitation programs can advantage the motor learning process and, at the same time, providing a direct feedback (e.g., visual, auditory) about performance level can increase patient interest and motivation [44, 48]. A new trend is the use of smartphone as monitoring systems or user-interface [53, 76, 84]. Implementation of suitable application (i.e., App) can provide a direct feedback to the patients and therapists about the progress in motor performance [26]. Gathered data could be remotely evaluated by the therapists [64]. Remote monitoring can provide useful information about patients’ status at every stage of rehabilitation pathway and, at the same time, it implies a greater centralization of patients role in the management of their own health associated to a more direct clinician control [101]. A typical architecture of remote monitoring systems includes: *i)* wearable sensing unit to gather movements data; *ii)* data storage and management in cloud computing; *iii)* software to analyse data and extract relevant clinical parameters [58, 66]. This approach implies collection of big amounts of data regarding personal information that requires ethical considerations and the definition of legal responsibility [102].

Most of the reviewed articles limited the application of wearable systems in short-time session for shoulder motion evaluation; only few studies performed longer monitoring periods of ADLs until 7 or 11 monitoring hours of 1 day [5, 6, 34].

## Conclusion

This review reveals that wearable systems are becoming an efficient and promising tool to evaluate shoulder health after neurological trauma or musculoskeletal injuries. Wearable systems have the potential to provide quantitative and meaningful clinical information about movement quality and progress in a rehabilitation pathway. The magneto-inertial measurements systems resulted the most used in clinical and research settings, followed by the growing application of smart-textiles for joint angles assessment. Despite of the accuracy of the current wearable systems in shoulder kinematics assessment, additional investigation needs to be executed to ensure long-term applicability in clinical settings and rehabilitation.

## Data Availability

The datasets used and/or analysed during the current study available from the corresponding author on reasonable request.

## Abbreviations

AB-AD: abduction-adduction
AC: acromioclavicular
CMS: Constant-Murley score
DASH: Disability of the Arm, Shoulder, and Hand
DOFs: degrees of freedom
EMG: electromyography
ER: external rotation
FLX-EXT: flexion-extension
GH: glenohumeral
HT: humerothoracic
IE: internal rotation
IER: internal-external rotation
IMU: inertial measurement unit
KF: Kalman filter
MEMS: micro-electro-mechanical systems
M-IMU: magnetic and inertial measurement unit
OLE: Optical Linear Encoder
PRISMA: Preferred Reporting Items for Systematic Reviews and Meta-Analyses
RC: rotator cuff
RMSE: root mean square error
ROM: range of motion
RTSA: Reverse Total Shoulder Arthroplasty
SC: sternoclavicular
SST: Simple Shoulder test
ST: scapulothoracic
TSA: Total Shoulder Arthroplasty
VAS: Visual Analogue Scale
